# Large Language Model–Powered 3D Avatars to Train Shared Decision-Making in Undergraduate Medical Students: Design and Mixed Methods Acceptance Study

**DOI:** 10.2196/102449

**Published:** 2026-10-27

**Authors:** Pieter Van Bostraeten, Lien Mertens, Jasmien Jaeken, Jeroen Bossen, Jaan Toelen, Geertruida Bekkering, Nicolas Delvaux, Bert Aertgeerts

**Affiliations:** 1 Academic Center for General Practice Department of Public Health and Primary Care KU Leuven Leuven, Flanders Belgium; 2 Department of Family Medicine Maastricht University Maastricht, Limburg The Netherlands; 3 Department of Development and Regeneration KU Leuven Leuven, Flanders Belgium; 4 Department of Orthopedics Universitair Ziekenhuis Leuven Leuven, Flanders Belgium; 5 Department of Pediatrics Universitair Ziekenhuis Leuven Leuven, Flanders Belgium

**Keywords:** shared decision-making, avatar, virtual patient, large language model, artificial intelligence, undergraduate medical education, simulation-based education, technology acceptance model, communication skills, medical education

## Abstract

**Background:**

Shared decision-making (SDM) is a core competency in patient-centered care, yet it is challenging for physicians. Simulation-based education with standardized patients (SPs) offers valuable practice opportunities for undergraduate medical education (UME), but is limited by cost, scalability, and variable feedback quality. Advances in large language model (LLM) technology and virtual patient (VP) design present a promising pathway toward accessible, realistic, and high-quality SDM skill training.

**Objective:**

This study aimed to develop LLM-powered, real-time interactive 3D avatars for SDM training in UME and to evaluate their acceptance among undergraduate medical students.

**Methods:**

We conducted a descriptive, cross-sectional mixed methods study at KU Leuven, Belgium (November-December 2025). The avatar system was developed through an iterative, interdisciplinary collaboration between medical educators, SDM experts, software developers, user experience designers, and AI engineers, grounded in prior work on physicians’ educational needs and SDM learning mechanisms. Development comprised prototype case authoring, usability testing with medical students, and iterative refinement. The final system is a web-based platform featuring real-time, LLM-powered spoken dialogue with diverse, photorealistic 3D patient avatars; layered prompts configuring avatar personality, clinical scenario, and difficulty; an educator panel for case authoring; and automated AI-generated formative feedback structured around 6 phases of SDM. After using the system for 1 month, a convenience sample of UME students completed a 9-item, 5-point Likert scale questionnaire grounded in the technology acceptance model (TAM), assessing perceived usefulness, perceived ease of use, and intention to use. Optional open-text responses were narratively analyzed to triangulate these findings.

**Results:**

Of 25 recruited participants, 24 were included in the analysis. Internal consistency was acceptable for perceived usefulness (α=0.76) and intention to use (α=0.78), but not for perceived ease of use (α=0.69). All construct means exceeded the scale midpoint of 3, suggesting high overall acceptance. Students rated perceived usefulness highest (mean 4.43, SD 0.50), followed by intention to use (mean 4.42, SD 0.59), and perceived ease of use was the lowest (mean 3.81, SD 0.68). Fourteen participants provided open-text responses. These highlighted the value of a safe, low-pressure environment for practicing communication without patients or peers present; reported mixed experiences with avatar realism, with reservations directed at behavioral rather than physical realism; and identified technical difficulties (microphone issues, freezing, and crashes) as the most frequent area for improvement.

**Conclusions:**

LLM-powered, real-time interactive 3D avatars are feasible and well-accepted tools for SDM training in UME. Students found the system educationally valuable and intended to use it again. Technical reliability and behavioral realism should be prioritized in future development. The modular architecture and automated feedback pipeline support scalable deployment across disciplines, institutions, and languages. Larger, multi-institutional studies with objective learning outcome measures are needed to establish efficacy.

## Introduction

Shared decision-making (SDM) is a collaborative process in which clinicians and patients make health decisions together, integrating medical evidence with patient preferences [1]. SDM has been linked to improved patient satisfaction, treatment adherence, knowledge, and health outcomes, while reducing decisional conflict [2]. SDM remains challenging for physicians because it demands the integration of multiple competencies: medical knowledge to explain options accurately, communication skills to convey benefits and risks and elicit patient values, and the attitude of viewing patients as equal partners in decision-making [3]. This integration must be flexibly applied across diverse clinical situations, each with varying degrees of uncertainty, emotional complexity, and patient readiness to engage.

Physicians voice the need for training in SDM, starting as early as undergraduate medical education (UME) [4,5]. Such training programs exist, typically combining didactic teaching with role play [6,7]. Besides attention to theory and professional identity formation, training requires skill integration through deliberate practice [8]. Simulation-based education, particularly with standardized patients (SPs), could provide the right learning conditions for this, especially in UME where real patient contact is often scarce.

SPs can be trained to portray a wide range of cases, provide predictable and repeatable scenarios, and give targeted feedback in a safe, learner-centered environment [9]. However, in reality, traditional training using SPs also has limitations: it is resource-intensive, repeatable practice is limited due to scheduling and cost, and feedback can be of variable quality [9,10]. These barriers restrict equitable, scalable access to SDM training and emphasize the need for innovative approaches that support accessible, repeatable, and high-quality practice.

In the last decade, virtual patients (VPs) have gained popularity in simulation-based training [11]. VPs are computer programs that simulate clinical scenarios in which learners take on the role of a health care provider to perform clinical tasks [12]. While being more accessible and customizable, they address limitations of traditional SP encounters, such as cost and logistical complexity [11,13]. There is some evidence that VPs could be as effective as traditional education for knowledge outcomes and may be even more effective for skills outcomes [14].

However, students feel negatively about VPs when they perceive them to be unrealistic or limited in natural responses [15]. These shortcomings may stem from early VP designs that relied on rigid, rule-based algorithms, scripted decision trees, or limited conversational freedom such as having to select from multiple choice options. Recent advances in AI and, more specifically, the use of large language models (LLMs) in VPs may support more natural, open-ended conversations, improving realism and learner engagement [16]. LLM-powered VPs have shown a “very good approximation of a real conversation,” and they can authentically portray different patient preferences [17]. Moreover, LLM-generated personalized feedback may be comparable to that provided by a trained clinician-supervisor [17].

Given the need for scalable, realistic, and accessible SDM skill training in UME, LLM-powered VPs offer a promising solution. To explore this potential, we developed an LLM-powered, real-time interactive 3D avatar system designed to support SDM skill training. We evaluated this avatar system from the learner’s perspective using the technology acceptance model (TAM) as an evaluative framework [18]. Specifically, we aimed to assess undergraduate medical students’ perceived usefulness, perceived ease of use, and intention to use the avatar system after hands-on practice, generating initial evidence on the feasibility and acceptance of LLM-driven avatar simulations in UME.

## Methods

### Study Design and Setting

We used a descriptive, cross-sectional mixed methods design to develop and evaluate an LLM-powered, real-time interactive 3D avatar system for medical students to practice SDM skills. The study was conducted at KU Leuven, Belgium. System development began in December 2022, and the student intervention was conducted from November to December 2025. Reporting of this study was guided by the Good Reporting of A Mixed Methods Study (GRAMMS) framework [19]; the completed checklist is provided in Multimedia Appendix 1.

### Development of the LLM-Powered Avatar System

#### Theoretical Foundations and Educational Embedding

The development of the avatar system was grounded in earlier theoretical work. First, we explored the educational needs of physicians and residents regarding SDM [5]. We learned that physicians need multiple skills-based learning opportunities, with feedback, in a supportive and safe learning environment. Specifically, they need training in communication skills, decision aid usage, challenging situations, and getting to know the patients’ perspectives.

Second, we conducted a realist review to understand how, why, and in what contexts educational interventions on SDM work in UME [8]. By mapping context-mechanism-outcome configurations in published SDM interventions, our review provided insights into the conditions under which SDM learning is most effectively supported. The review emphasized the need for integrating theory and skills through repeated practice in safe and supportive environments, as well as attention to theoretical learning and professional identity formation. Learning should be scaffolded, contextualized, and aligned with learners’ future clinical practice. Additionally, SDM education should promote self-regulated learning.

The avatar exercise was conceptually grounded in the 3-talk model of SDM by Elwyn et al [20], which structures the consultation into team talk, option talk, and decision talk, and was further informed by the behavioral indicators described by Stiggelbout et al [1] and the entrustable professional activities for SDM proposed by Baghus et al [21]. These frameworks guided the design of the clinical cases, the layered prompt architecture, and the criteria used for automated feedback generation.

These insights directly informed the educational embedding of the avatar exercise, which was designed as a structured, low-risk environment to support the integration of theory and practice. The exercise was integrated into a broader educational program comprising (1) a preparatory e-learning module to establish foundational knowledge about SDM, (2) the avatar interaction for experiential skill practice, and (3) a workshop to support reflection and professional identity formation. This study evaluated only the avatar exercise (2), while the broader program (1, 2, and 3) was studied separately.

#### Team Composition and Collaboration

The avatar system was developed through a collaboration between the Academic Center for General Practice (KU Leuven) and Raccoons, a Belgian company specialized in developing AI solutions. The interdisciplinary team included medical educators, SDM experts, software developers, user experience designers, and AI engineers. Regular meetings facilitated co-creation across disciplines.

#### Development Activities

Development followed an iterative, multidisciplinary approach. The process started with a kickoff workshop involving the full team of clinicians, communication skills educators, researchers, and developers to align educational goals and technical design criteria. During this session, a shared understanding was established regarding the principles of SDM, communication skills training, and feedback practices.

To ground the design in authentic educational practice, a user experience designer observed one of our traditional communication skills training sessions in which medical students practice with simulated patients, while peers observe and provide feedback together with faculty.

The academic team subsequently developed several prototype cases, including examples of effective and suboptimal SDM conversations and corresponding examples of high-quality feedback [1,20,22]. These materials served as didactic reference points for dialogue behavior and automated feedback generation.

Next, user testing was conducted with medical students. First, we conducted moderated usability testing of the system’s interface (prior to implementation of the full real-time interactive avatar functionality) in a convenience sample of 8 medical students to explore user experience, interface clarity, and perceived usability based on a clickable prototype using Figma software. Second, a real-time interactive avatar prototype was tested in a convenience sample of 2 medical students to identify technical and interactional improvement points.

Finally, the functional avatar system underwent multiple rounds of iterative refinement by the multidisciplinary team. Testing and revision occurred both during weekly team meetings and through asynchronous review cycles, with continuous adjustments until consensus was reached on educational and technical adequacy.

### Final System

The final product is a web-based VP system that delivers a real-time, spoken conversational practice opportunity for medical students in their native language (Dutch). Learners interact with AI-driven avatars through natural spoken dialogue or typing. Students can access the system on a standard desktop or laptop computer with a microphone. No local installation is required because computationally intensive processing and rendering occur server-side and are streamed to the learner’s browser as live video.

### System Overview

The system features a secure, individualized login system, ensuring that each student's progress and data remain private. Upon authentication, students are presented with a case overview interface displaying available clinical cases, each accompanied by a brief title, short description, difficulty level (easy, medium, or hard), and completion status (Figure 1). Cases can be sorted alphabetically or by difficulty, and previous feedback can be accessed directly from this page. Upon selecting a case, students are directed to a case preparation interface presenting a detailed description of the scenario, the learning objectives, the treatment options to be discussed, and a decision aid designed to support the upcoming conversation. Once the exercise is initiated, students are immersed in a first-person consultation environment rendered as a realistic clinical setting, in which they interact with the avatar via typed or spoken input (Figure 2). Throughout the exercise, the case description, learning objectives, and decision aid remain accessible without interrupting the session. Upon terminating the interaction, students are directed to a feedback interface displaying a full transcript of the consultation alongside AI-generated, personalized feedback (Figure 3). From this screen, students can review feedback from prior attempts and navigate back to the main case overview page.

**Figure 1:**
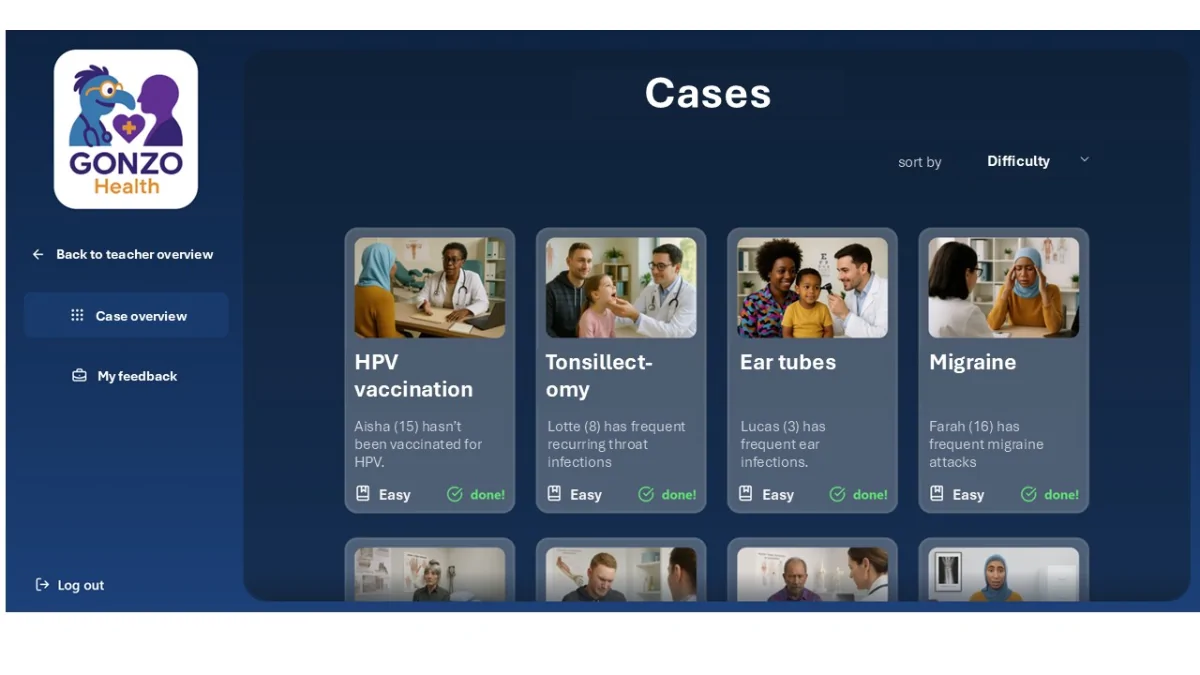
Case overview interface of the avatar system, listing the available shared decision-making cases with title, short description, difficulty level, and completion status. HPV: human papillomavirus.

**Figure 2:**
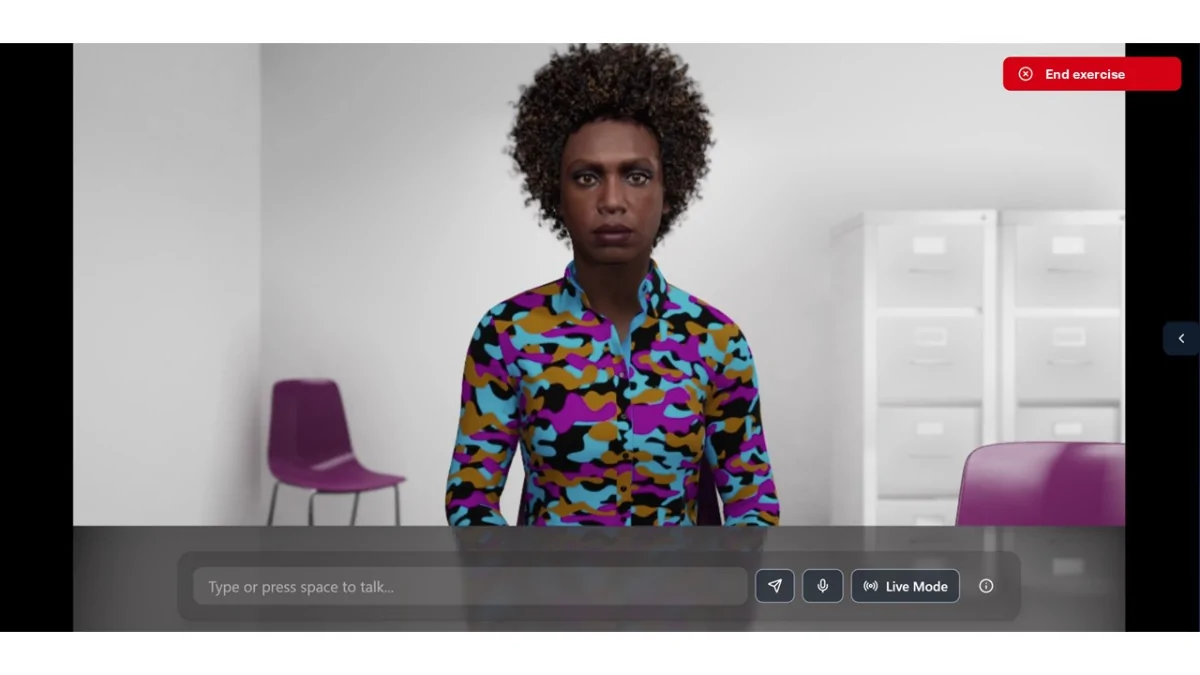
Case exercise interface, showing the first-person consultation environment in which the student interacts with the avatar through typed or spoken input.

**Figure 3:**
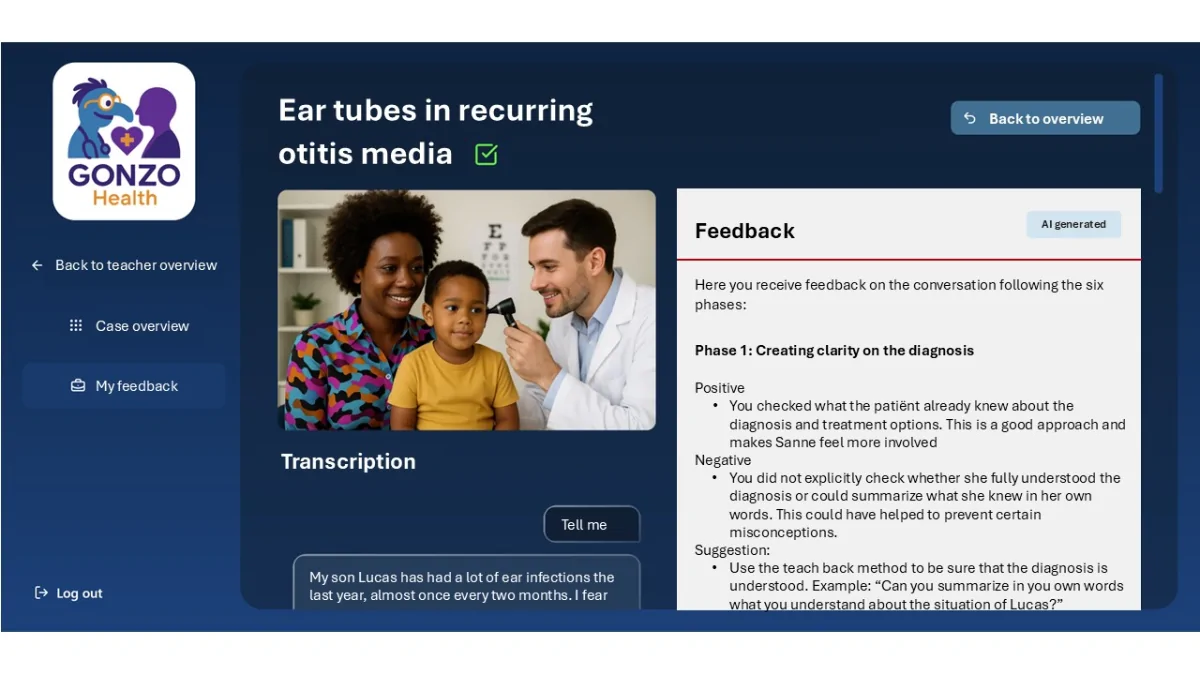
Feedback interface, showing the verbatim consultation transcript alongside the automated, AI-generated formative feedback.

### Real-Time Interactive Avatar Pipeline

The avatar exercise uses the following conversational pipeline (Figure 4): (1) the student speaks to the avatar, (2) speech is converted to text, (3) the language model generates a response based on the provided context (layered prompts, see later), (4) the response is converted back to speech, and (5) the avatar delivers the response with synchronized facial and body behavior. Speech-to-text is performed using browser-based speech recognition for responsiveness; text-to-speech uses ElevenLabs to provide natural-sounding, multilingual voices that could be tuned to different patient types (eg, age, emotion, and tone) or even languages.

**Figure 4:**
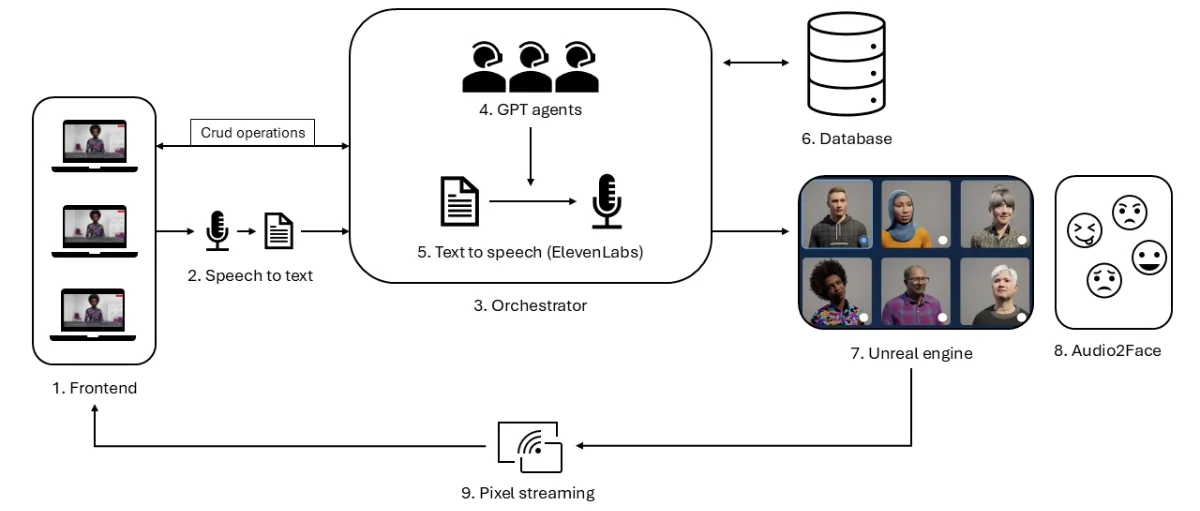
Real-time interactive avatar pipeline, from student speech input through speech-to-text conversion, large language model response generation, text-to-speech conversion, and synchronized avatar delivery.

For dialogue generation, we used OpenAI GPT models via Azure OpenAI hosted in European data centers. Specifically, GPT-4o was used to prioritize responsiveness and natural conversation. Student conversations are not used to train the models; the system uses only the immediate input to generate responses.

### Diverse and Configurable Avatars

To support realism and representation, we developed multiple 3D patient avatars reflecting diversity in age, appearance, skin tone, and cultural background after consulting a Belgian patient representation organization (Figure 5). Avatars were created using MetaHuman technology (Unreal Engine) to achieve high visual fidelity, a design choice intended to enhance credibility and immersion during communication skills training. Avatars are configurable (eg, age, hair, skin tone, and facial features) and can be extended with custom elements such as accessories and cultural markers (eg, a headscarf).

Avatar nonverbal behavior was designed to match clinical conversations. Body language was captured using a motion-capture suit (Rokoko), and lip and facial movements were generated using Audio2Face (NVIDIA) to synchronize articulation and expression with the generated speech. Custom-recorded subtle behaviors (eg, breathing, small head movements, and worried affect) were used to better approximate patient communication cues.

**Figure 5:**
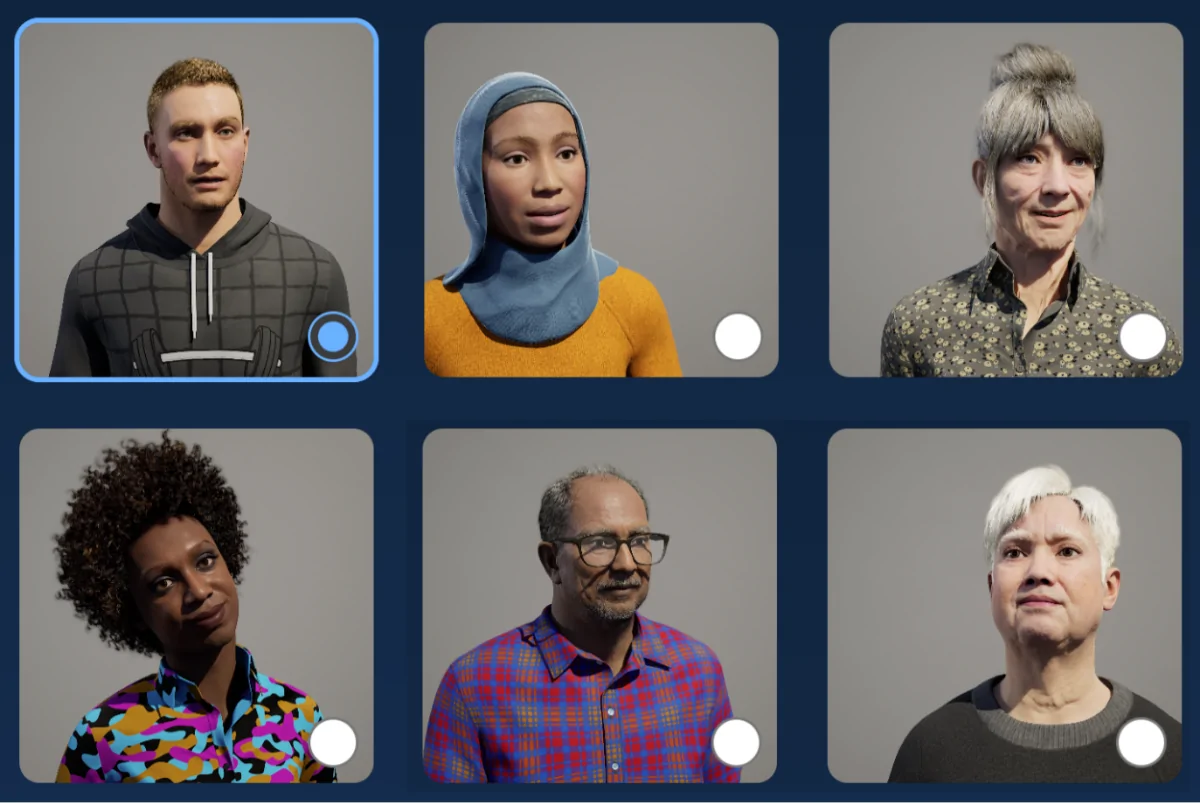
Diverse set of 3D patient avatars, reflecting variation in age, appearance, skin tone, and cultural background.

### Authentic Clinical Cases with Increasing Complexity

Cases represent preference-sensitive clinical decisions suitable for SDM, such as recurrent otitis media in children, carpal tunnel syndrome, and human papillomavirus (HPV) vaccination. Before each encounter, students receive (1) a brief case framing with conversation goals and (2) access to a built-in decision aid that could be consulted during the dialogue. Decision aids were either sourced from Thuisarts [23], a patient information platform developed and endorsed by the Dutch College of General Practitioners (Nederlands Huisartsen Genootschap), or were developed in collaboration with clinical staff and consisted of a plain-text overview of the available treatment options and their respective benefits and risks. Pediatric and orthopedic cases were developed in collaboration with clinical faculty. These disciplines were chosen pragmatically, as faculty in both disciplines were interested and willing to implement the avatars within their courses in the future.

Each case is built from multiple layered prompt components that together shape how the avatar behaves during the exercise. The first layer defines the avatar’s personality: age, background, tone of voice, emotional baseline, and conversational style. This personality is consistent across different encounters and gives the avatar a stable, recognizable character. In addition, a clinical scenario is added. It describes the patient’s medical situation alongside personal dimensions such as the patient’s own ideas about their condition, their concerns, their expectations of the consultation, and their preferences. Together, these elements reflect the biopsychosocial complexity that learners are expected to navigate. A third layer introduces difficulty, modulating the encounter through additional behavioral or communicative challenges such as decisional uncertainty, emotional distress, or unrealistic expectations. Difficulty levels can be ordered and labelled (eg, easy, medium, and hard), allowing educators to scaffold exposure. Finally, each case can be enriched with explicit learning goals that define the conversational objectives. At runtime, the backend assembles the relevant components into a single, coherent instruction that is passed to the underlying AI model, ensuring that the avatar's responses remain contextually consistent throughout the encounter.

### Transcripts and Feedback

After each encounter, students automatically receive a verbatim transcript of the full dialogue and structured, AI-generated formative feedback. Feedback is generated using a separate LLM (GPT-o3 mini), selected for its stronger analytical and reasoning capabilities.

Feedback generation is guided by a predefined evaluation prompt that operationalizes SDM and clinical communication into observable behavioral indicators [1,20,21]. These indicators include, among others: inviting the patient to participate in the decision-making process, provision of balanced information about options (benefits and risks), eliciting values and concerns, supporting deliberation, and arriving at a shared decision. In addition, the prompt incorporated established feedback principles from clinical communication education (eg, specificity, behavior-focused comments, balanced reinforcement and improvement points, and actionable suggestions for future encounters) [22]. The model was instructed to structure feedback into clearly delineated sections (strengths, areas for improvement, concrete suggestions, and a summary). To prevent the model from generating inaccurate or fabricated feedback, we narrowed its scope by explicitly withholding feedback when conversations were too short and limiting feedback to specific criteria such as the 6 phases of SDM, excluding body language. An anonymized and translated example of a student-avatar dialogue with the corresponding automated feedback is provided in Multimedia Appendix 2. The behavioral indicators on which feedback is given are summarized in Textbox 1.

**Shared decision-making behavioral indicators and feedback structure used in the automated feedback generation system.**

The automated feedback system evaluates each student-patient conversation along 6 sequential phases of shared decision-making (SDM), grounded in established SDM frameworks [1,20,21]. Feedback is withheld when the conversation is too short to assess (eg, limited to a greeting). Feedback is restricted to verbal content only. Body language and tone are explicitly excluded. For each phase, the system provides balanced feedback identifying both strengths and areas for improvement, accompanied by concrete suggestions.

- Phase 1: clarifying the diagnosis. The student verifies the patient’s understanding of the diagnosis and, where understanding is insufficient, explains it in plain language and checks comprehension using the teach-back method.
- Phase 2: announcing the decision-making. The student makes clear that a decision needs to be made, signals that multiple options exist, and explicitly invites the patient to participate in the decision.
- Phase 3: information exchange. The student assesses the patient’s existing knowledge, explains all relevant treatment options (including watchful waiting where applicable) with their benefits and risks in plain language, and verifies comprehension using the teach-back method.
- Phase 4: exploring values and preferences. The student actively elicits what matters most to the patient, explores how the options fit into the patient’s daily life and values, summarizes the patient’s preferences, and checks whether relevant third parties (eg, family) should be involved.
- Phase 5: arriving at a shared decision. The student summarizes the options and the patient’s stated preferences, actively involves the patient in the final choice, clearly articulates the agreed decision or defers it where appropriate, and confirms the patient’s understanding and agreement.
- Phase 6: closing the consultation. The student clarifies that the decision can be reconsidered, makes concrete follow-up arrangements, provides written information where relevant, offers time for reflection, and explicitly invites final questions before closing empathically.
- General communication skills. In addition to the 6 SDM phases, the system provides feedback on general communication, including use of plain language, reflective and empathic listening, nonjudgmental responses to patient preferences and emotions, and creation of a safe and open conversational atmosphere.

### Case Authoring and Configuration Interface (Educator Panel)

As cases are modular, new scenarios can be added without technical rework of the system. The system includes a prototype, web-based educator interface that enables faculty to independently create and configure case scenarios. This interface is still in development and was not used for this study. Within this interface, educators can pick an existing avatar and give it personality traits, write a case scenario, select differing difficulty grades, and provide objectives and feedback rules. Educators can also add a decision aid through this interface.

### Infrastructure, Scalability, and Data Governance

Rendering and heavy computation are performed on cloud infrastructure. Each concurrent user session temporarily provisions a GPU-enabled cloud server. Therefore, operational cost scales with simultaneous usage. This architecture was an explicit trade-off favoring realism and quality, with future optimizations anticipated as streaming and rendering technologies evolve.

Regarding governance, KU Leuven acted as data controller and the technology partner as processor, covered by a formal data processing agreement. Data were handled within the agreed educational and research context and were not reused for model training; processing occurred through Azure OpenAI in European Union data centers to support privacy and compliance requirements.

### Participants and Recruitment

We recruited a convenience sample of undergraduate medical students enrolled in the first or second year of the Master of Medicine program at the Faculty of Medicine, KU Leuven. The educational program was not embedded in a specific course; participation was entirely voluntary, and no exclusion criteria were applied. Email invitations were sent by the faculty on behalf of the research team to all students enrolled in the first or second Master year, followed by 1 reminder, over a period of 3 weeks. The invitation described the content of the training program, the estimated time investment, the voluntary nature of participation, and the contact details of the research team; the same information was provided through 2 in-class presentations and flyers. Students who contacted the research team subsequently received an information letter detailing the educational program, the data collection, the processing of their personal data and their rights, and were asked to complete an informed consent form. Because of the low response, all students who expressed interest were enrolled.

### Procedure

Participants completed the full educational program (see earlier) and interacted with the avatars at their own pace, in their place and time of choice during 4 weeks (November-December 2025). Half practiced with pediatric cases, and the other half with orthopedic cases. In order to have 1 concurrent user at a time, students needed to pick available time slots. Students received a very brief instruction prior to starting. Students were free to use the system as they liked. Upon completion of the educational program, all students filled out a questionnaire (see Measures).

### Measures

We developed and administered a postuse questionnaire grounded in the TAM [18]. TAM is an established information systems theory describing how users come to accept and use a new technology. According to this theory, perceived usefulness and perceived ease of use influence users’ attitudes and behavioral intention to use a system, which in turn predicts actual system use.

The questionnaire assessed three TAM-derived constructs: (1) perceived usefulness, reflecting the extent to which students believe that using the avatar system enhanced their SDM skills, understanding, and training; (2) perceived ease of use, reflecting the extent to which students experienced the avatar system as easy, intuitive, and effortless to use; and (3) intention to use, reflecting the extent to which students intend to use the avatar system again in the future and recommend it to others as a learning tool.

Each construct consists of 3 items, resulting in a total of 9 items (Textbox 2). All items were rated on a 5-point Likert scale ranging from 1 (“strongly disagree”) to 5 (“strongly agree”). Construct scores were calculated by averaging item responses within each construct, with higher scores indicating greater perceived usefulness, ease of use, or intention to use.

In addition, we included optional open-text fields to allow students to provide qualitative feedback on their experience with the avatars.

**Items of the postuse questionnaire assessing perceived usefulness, perceived ease of use, and intention to use of the avatar system among undergraduate medical students.**

**Perceived usefulness**

- Item 1: The avatars helped me improve my shared decision-making skills.
- Item 2: The avatars were valuable for my medical training.
- Item 3: Thanks to the avatars, I understand the concept of shared decision-making better.

**Perceived ease of use**

- Item 4: The avatars were easy to use.
- Item 5: The interaction with the avatars was smooth and intuitive.
- Item 6: I had no trouble understanding how to use the avatars.

**Intention to use**

- Item 7: I would like to use the avatars again in the future to learn.
- Item 8: I would recommend the avatars to other students to learn.
- Item 9: I see myself working with these kinds of digital learning tools more often.

### Statistical Analysis

We performed all statistical analyses using JASP (version 0.95.4; University of Amsterdam). To address the research questions, we computed descriptive statistics (means and standard deviations) for each individual questionnaire item as well as for the composite construct scores. To evaluate the psychometric properties of the questionnaire, we assessed the internal consistency of the 3 constructs using Cronbach alpha, with a threshold of .70 considered acceptable [24]. To compare construct scores between the students who practiced with pediatric cases and those who practiced with orthopedic cases, we first assessed normality using the Shapiro-Wilk test, which indicated deviation from normality. Therefore, a 2-tailed Mann-Whitney *U* was used as a nonparametric alternative to the independent samples *t* test.

Open-text responses were used to triangulate the questionnaire findings. Two researchers (PVB and LM) read all responses and jointly identified recurring points, including critical remarks, which were narratively summarized with illustrative quotes where appropriate. Given the small number of responses and their brevity, findings were used to provide additional context rather than constituting a standalone qualitative analysis.

### Ethical Considerations

The Social and Societal Ethics Committee of KU Leuven approved this study (G-2024-7948, G-2025-9755). All participants provided written or digital informed consent before the start of the educational activity. All data were pseudonymized using a key accessible only to the first author (PVB); this key was deleted after analysis, so that the data underlying this report are fully anonymized, and participants can no longer be reidentified. Data were stored securely in accordance with institutional data protection guidelines and were used for research purposes only. Participants received 2 cinema tickets as compensation for their participation.

## Results

### Participant Characteristics

We recruited 25 participants, of whom 1 participant was excluded for not starting any practice sessions, resulting in a final sample of 24 participants (n=24). Table 1 presents the demographic characteristics.

**Table 1:** Demographic characteristics of the undergraduate medical students who practiced shared decision-making with the avatar system (n=24).

Characteristics and categories			Participants, n (%)
Sex			
	Female	17 (70.8)	
	Male	7 (29.2)	
Age (years)			
	20-21	9 (37.5)	
	22-23	14 (58.3)	
	24-25	1 (4.2)	
Native language			
	Dutch	24 (100)	
Year of study			
	4th	7 (29.2)	
	5th	17 (70.8)	
Previous training in SDM^a^			
	Yes	3 (12.5)	
	No	21 (87.5)	

^a^SDM: shared decision-making.

### Psychometric Properties of the Questionnaire

Results indicated acceptable reliability for “perceived usefulness” (α=0.756) and “intention to use” (α=0.778). The “perceived ease of use” construct did not reach this threshold (α=0.690), suggesting that the items within this construct may not sufficiently measure the same underlying concept.

### Questionnaire Results

All 24 students completed all 9 items of the 5-point Likert scale questionnaire after practicing with the avatar system. Table 2 presents the results.

Respondents scored highest on “perceived usefulness” (mean 4.431, SD 0.496) and lowest on “perceived ease of use” (mean 3.806, SD 0.680). All construct means were above the scale midpoint of 3.0.

No statistically significant differences were found between the 2 groups for any of the 3 constructs (all *P*>.05), suggesting that the type of cases did not significantly influence participants’ ratings of perceived usefulness, perceived ease of use, or intention to use (Multimedia Appendix 3).

**Table 2:** Descriptive statistics per questionnaire item and technology acceptance model construct among undergraduate medical students after practicing with the avatar system (n=24).

Construct and items			Mean (SD)
Perceived usefulness			4.43 (0.50)
	U1	4.58 (0.58)	
	U2	4.63 (0.58)	
	U3	4.08 (0.65)	
Perceived ease of use			3.81 (0.68)
	E1	3.79 (1.02)	
	E2	3.42 (0.65)	
	E3	4.21 (0.88)	
Intention to use			4.42 (0.59)
	I1	4.46 (0.66)	
	I2	4.63 (0.58)	
	I3	4.17 (0.87)	

### Qualitative Feedback

Of the 24 participants, 14 provided a written response. Several recurring themes emerged.

Multiple participants described the avatar system as a valuable tool for practicing communication skills at their own pace. Two respondents specifically highlighted the benefit of practicing without the external pressure of patients or peers, resulting in a safe learning environment that still felt authentic. One participant wrote the following:

Very convenient for practicing communication without the pressure of patients, or without it feeling like a performance with fellow students. Convenient to actually try out practical sentences that you can later apply in practice.

Another added:

It seems like a very useful tool for medical students because it helps you gain more confidence in your communication without having to do it in front of a class of students.

Only 1 student commented on the automated feedback delivery, writing that it occasionally flagged SDM components as insufficient despite having addressed them.

Technical difficulties were the most frequently mentioned concern. Five respondents reported problems with speech conversation specifically. One participant explained:

The consultations with the avatars did not always go smoothly. The avatars worked fine when I typed the conversations, but I found that the 'live' or 'microphone' function did not always capture the conversation well.

Another noted that it was “best to speak very slowly so that everything is clearly picked up.” Three participants experienced the exercise freezing or crashing, with one reporting the following:

They kept hanging for a very long time or the program froze, and I also did not receive feedback on several occasions.

One respondent noted that it was initially unclear where to find the feedback after a session.

Opinions regarding the realism of the avatars were mixed. One participant described the experience as immersive:

In the beginning I doubted whether it would be realistic and would work, but actually it went very smoothly for me. It really felt as if I was speaking with a patient.

Conversely, 3 others felt the avatars lacked realism to a certain extent. One wrote that “the avatars were personally just a bit too far from reality. Too understanding, too compliant, too concrete in their answers.” One participant specifically mentioned the absence of facial expressions as a limitation, while another expressed optimism that this would improve as AI technology develops. One participant mentioned that being able to speak to the avatars made the exercise more realistic.

Finally, individual participants offered suggestions for improvement, including expanding the tool to other conversation skills, offering different patient cases at each difficulty level to avoid repetition of treatment options, and providing clearer upfront instructions on where to access feedback.

## Discussion

### Summary of Findings

This study presents the design and development of LLM-powered, real-time interactive 3D avatars to support SDM skill training, and evaluates its perceived usefulness, perceived ease of use, and intention to use among undergraduate medical students. Overall, the system was received positively. Perceived usefulness and intention to use were rated highest, while ease of use scored somewhat lower, attributable to technical difficulties encountered using the system. No significant differences were found between students practicing with pediatric versus orthopedic cases.

### Interpretation of Findings and Comparison to Literature

Medical students scored positively on all 3 TAM constructs, suggesting high overall acceptance of the avatars. This aligns with TAM theory, which posits that perceived usefulness and perceived ease of use are determinants of behavioral intention [18]. Earlier studies applying TAM to virtual simulation in health care education similarly report high scores on perceived usefulness and behavioral intention, with ease of use typically rated somewhat lower [25-28]. Across these studies, perceived usefulness tends to be the strongest and most consistent predictor of behavioral intention, confirming findings of broader simulation literature stating that learners recognize the educational value of simulation-based tools even when technical limitations exist [29]. According to our qualitative findings, great value seems to be in allowing students to practice without the pressure of patients or fellow students present. Indeed, students find the opportunity to practice in a safe, low-pressure environment as a key driver of positive attitudes toward VPs [15]. On the other hand, the lower ease of use scores across studies, including our own, tend to reflect specific technical shortcomings that lead to frustration, rather than resistance to the technology itself [15].

Although students rated the avatars highly, qualitative comments from a subset of participants raise concerns that merit further exploration. A few participants expressed reservations about the realism of the avatars and their responses. As physical resemblance may support learner engagement and believability of the exercise, we invested substantially in physical avatar realism through MetaHuman technology, motion capture, and facial synchronization. However, our qualitative observations align with broader simulation literature, increasingly arguing that educational effectiveness of simulation depends less on physical resemblance than on functional task alignment, being the degree to which the simulator’s functional properties correspond to the demands of real clinical tasks [30,31]. High visual fidelity may even increase cognitive load by directing learner attention toward irrelevant features, and near-realistic representation additionally risks the uncanny valley effect, whereby subtle imperfections in an otherwise lifelike avatar elicit discomfort rather than affinity [30,32,33]. Our participants’ concerns point to gaps in semantical realism (the information structure of the interaction) and phenomenal realism (the subjective experience of the scenario), such as the absence of facial expressions and realistic communicative behaviors, rather than physical realism [34]. Real-world SDM consultations are indeed dependent on reading nonverbal cues such as hesitation, distress, and confusion, and require students to navigate a variety of patient behaviors including anxiety, misinformation, reluctance, and emotional complexity. While we intended to account for the complexities of SDM through various measures, these elements may not yet be sufficiently perceptible at the level of interaction, warranting refinement in future development.

Our system development contributes to a rapidly expanding body of work on LLM-powered VPs in medical education. A recent scoping review identified 28 such systems, of which 92.9% were published within the past 2 years [16]. Our system differs from many existing systems in several respects. First, the majority of identified systems operated as text-based chatbots, whereas our system supports real-time spoken dialogue. Although rigorous comparative evidence is missing, voice-based avatar simulations seem to yield greater self-reported learning benefits than text-based chatbots, as they are more authentic and support conveying social and emotional cues [35,36]. Second, most systems targeted history-taking or clinical reasoning while ours is specifically designed for SDM training. SDM places different demands on the learner: the student must present options, respond to patient values and emotions, and co-construct a decision, hence moving beyond information delivery toward relationship-centered, value-sensitive collaboration [1,37]. The only identified precedent for VP-based SDM training used a multiple-choice format requiring students to recognize correct SDM steps [38,39]. Free spoken dialogue more closely mirrors the communicative demands of real SDM consultations. Third, only a minority of systems used automated feedback on learner performance. Feedback is a crucial driver of learning because it allows learners to confirm, correct, or refine their understanding and strategies by supplementing their own self-monitoring of performance relative to goals [40]. Emerging evidence suggests that automated, LLM-generated feedback can fulfill this function effectively in clinical education settings [41,42]. LLM-based systems can provide specific, actionable feedback with quality ratings similar to human raters, though they are sometimes overly positive or missing key weaknesses [17,41]. Students do not always rate AI-generated feedback similar to human tutor feedback, yet see additional value due to its immediacy, breadth, and scalability, suggesting that a hybrid model combining both may be more effective than either alone [43].

### Strengths and Limitations

This study has several strengths. The development of the system was grounded in prior theoretical work on educational needs and SDM learning mechanisms. This distinguishes it from the majority of existing GenAI-supported VP systems, which lack such theoretical grounding [44]. Combined with an iterative, interdisciplinary development process, this theoretical foundation contributed to the credibility and applicability of the resulting system. The system’s modular, layered prompt architecture, the educator configuration interface, the automated feedback, and the diversity of the avatar set are design features with broad applicability beyond this study’s specific educational context. Notably, by integrating diverse patient representations, the system implicitly reinforces that the same quality standards in communication and SDM apply regardless of patient profile. The use of a validated theoretical framework (TAM) provided a structured basis for evaluating acceptance, with established precedent in medical education research. The mixed methods approach, combining Likert-scale scores with open-text qualitative feedback, allowed for triangulation of findings. Finally, the technology partner had no involvement in the analysis or interpretation of results, mitigating potential conflicts of interest.

Several limitations must be acknowledged. The sample was small, drawn from a single institution, and consisted exclusively of Dutch-speaking students, which substantially limits generalizability. As participation was voluntary and convenience-based, selection bias is plausible: students who enrolled may have had greater interest in technology, innovation, or communication training than their peers, potentially inflating acceptance scores. All outcome measures were self-reported, introducing risk of social desirability bias. The questionnaire was developed specifically for this study, drawing on the constructs and theoretical framework of TAM, but was not formally validated prior to its use, and the internal consistency of the ease-of-use subscale did not reach the accepted threshold. The system was evaluated as part of a broader educational program. While the questionnaire specifically targeted students’ perceptions of the avatar system, it cannot be excluded that the broader program context influenced how students experienced and rated the avatars. Finally, while TAM provided a practical and well-validated framework for this initial evaluation, its basic model applied here captures only perceived usefulness, perceived ease of use, and intention to use. It does not account for broader contextual factors, such as subjective norms, enjoyment, immersion, or pedagogical relevance, that may play an important role in shaping students’ acceptance of educational technology [45]. Additionally, TAM has been criticized for explaining a relatively modest proportion of variance in adoption behavior, with the gap between intention and actual use rarely interrogated [46]. Future studies would benefit from incorporating extended TAM constructs or complementary frameworks, and from measuring actual system use behavior alongside self-reported perceptions.

### Implications and Future Research

The findings of this study have several implications. First, they contribute to the growing body of evidence that LLM-based avatars are a feasible and accepted modality for communication skills training in UME, now including SDM training. The rapid proliferation of such systems underscores the importance of systematic evaluations that go beyond technical proof-of-concept to include structured learner acceptance data. By applying a validated theoretical framework and collecting both quantitative and qualitative data, this study contributes a replicable evaluation model to this emerging literature. Second, the system’s modular architecture and educator panel allow for case expansion without technical rework, making it potentially scalable to other clinical disciplines, institutions, and languages. The multilingual capability of the text-to-speech pipeline is a particular asset for international adoption. We are currently working on an improved version of the system, based on students’ and educators’ experiences. Third, the university-private partnership model underlying this system offers a template for future co-development efforts.

Several priorities emerge for future research. The system’s impact on actual learning transfer needs to be evaluated, assessed in real or simulated clinical encounters. Such studies should use validated SDM assessment instruments and include longer follow-up periods to examine retention. Relatedly, the quality and accuracy of the AI-generated feedback should be investigated against expert clinician feedback, for example by rating the student-avatar dialogues with validated observer-rated SDM instruments such as the OPTION scale [47] or Observer OPTION 5 [48] and comparing these ratings with the automated feedback. This is particularly relevant because the students in this study had no clinical SDM experience and were therefore not positioned to judge the accuracy or educational validity of the feedback they received. The avatar system should also be evaluated in larger, multi-institutional samples and with linguistically and culturally diverse student populations to assess generalizability. Improving the behavioral realism of the avatars beyond physical resemblance is a design priority identified by students in this study and supported by the broader VP literature. Finally, students suggested expanding the system to other communication exercises such as breaking bad news. Exploring whether the layered prompt architecture can be extended to such scenarios without loss of fidelity would be a valuable contribution to the field.

### Conclusions

This study presents the development and acceptance evaluation of LLM-powered, real-time interactive 3D avatars for SDM training in UME. Students rated the system highly on perceived usefulness and intention to use, supporting its feasibility and acceptance as an educational technology. Qualitative findings confirmed the value of a safe, autonomous practice environment, while highlighting technical issues and limited behavioral realism as priorities for further refinement. The system’s modular architecture and automated feedback pipeline allow for scalable deployment across clinical disciplines, institutions, and languages. Future research should assess actual learning transfer using validated SDM outcome measures, examine the accuracy of AI-generated feedback against expert standards, and evaluate the system in larger, more diverse student populations.

## Appendix

Multimedia Appendix 1 GRAMMS checklist.

Multimedia Appendix 2 Example of an anonymized and translated student–avatar dialogue with the corresponding automated feedback.

Multimedia Appendix 3 Comparison of construct scores between students who practiced with pediatric cases (n=11) and those who practiced with orthopedic cases (n=13).

## Abbreviations

GRAMMS: Good Reporting of A Mixed Methods Study
HPV: human papillomavirus
LLM: large language model
SDM: shared decision-making
SP: standardized patient
TAM: technology acceptance model
UME: undergraduate medical education
VP: virtual patient

## Data Availability

Data to support the findings of this study are either provided within the manuscript or supplementary materials, or are available from the corresponding author on reasonable request.
