# Supplementary material for: Large Language Model–Powered 3D Avatars to Train Shared Decision-Making in Undergraduate Medical Students: Design and Mixed Methods Acceptance Study
Source: JMIR Form Res. 2026 Oct 27;10:e102449. doi: 10.2196/102449 (PMC13752591; doi:10.2196/102449)
Supplement: Supplementary file 1 [file formative_v10i1e102449_app1.pdf]

Multimedia Appendix 1. Good Reporting of A Mixed Methods Study (GRAMMS) checklist

Manuscript: LLM-Powered 3D Avatars to Train Shared Decision Making in Undergraduate Medical Students: Design and Mixed Methods Acceptance Study

Guideline: O'Cathain A, Murphy E, Nicholl J. The quality of mixed methods studies in health services research. J Health Serv Res Policy. 2008;13(2):92-98. doi:10.1258/jhsrp.2007.007074

#	GRAMMS item	How it is addressed in this study	Location (page, lines)
**1**	Describe the justification for using a mixed methods approach to the research question.	The Technology Acceptance Model questionnaire quantified students' acceptance of the avatar system. Optional open-text responses were added to triangulate these scores and to provide context on students' experiences that the Likert-scale items could not capture.	Methods 3.1: p5, L115–116<br>Methods 3.7: p16, L363–367<br>Strengths: p22, L518–519
**2**	Describe the design in terms of the purpose, priority and sequence of methods.	Purpose: triangulation of the questionnaire findings.<br>Priority: quantitative dominant; the qualitative data provide additional context rather than a standalone qualitative analysis.<br>Sequence: concurrent; both components were collected in the same post-use questionnaire after the practice period.	Methods 3.1: p5, L115–116<br>Methods 3.5: p15, L326–331<br>Methods 3.6: p16, L351–352<br>Methods 3.7: p16, L363–367
**3**	Describe each method in terms of sampling, data collection and analysis.	Sampling: one convenience sample of undergraduate medical students for both components (N=24); 14 participants provided open-text responses.<br>Data collection: a 9-item, 5-point Likert scale questionnaire based on the Technology Acceptance Model (Table 1), with optional open-text fields in the same questionnaire.<br>Analysis: quantitative data were analyzed in JASP (descriptive statistics, Cronbach's alpha, Shapiro-Wilk test, Mann-Whitney U test); open-text responses were read by two researchers, who jointly identified recurring points and summarized them narratively with illustrative quotes.	Sampling: p14–15, L313–324; p18, L404<br>Data collection: p15, L333–347; p16, L351–352<br>Analysis: p16, L354–367
**4**	Describe where integration has occurred, how it has occurred and who has participated in it.	Where: in the interpretation of the results (Discussion).<br>How: the open-text responses were used to triangulate and explain the questionnaire scores, for example the lower perceived ease of use and the reservations about avatar realism.<br>Who: the open-text responses were analyzed by two researchers (PVB, LM).	Methods 3.7: p16, L363–364<br>Discussion: p19–20, L443–444; p20, L456–461; p20–21, L462–481
**5**	Describe any limitation of one method associated with the presence of the other method.	Because the qualitative component consisted of optional, brief open-text responses embedded in the questionnaire, it was not suitable for a standalone qualitative analysis and was used only to provide additional context. Not all participants provided open-text responses (14 of 24).	Methods 3.7: p16, L365–367<br>Results 4.4: p18, L404

#	GRAMMS item	How it is addressed in this study	Location (page, lines)
**6**	Describe any insights gained from mixing or integrating methods.	The open-text responses attributed the lower perceived ease-of-use scores to technical difficulties, identified practicing in a safe, low-pressure environment as a key source of perceived usefulness, and showed that reservations about realism concerned behavioral rather than physical realism.	Discussion: p19–20, L443–444; p20, L456–461; p20–21, L462–481<br>Conclusion: p24, L578–580
