# Supplementary material for: Large Language Model–Powered 3D Avatars to Train Shared Decision-Making in Undergraduate Medical Students: Design and Mixed Methods Acceptance Study
Source: JMIR Form Res. 2026 Oct 27;10:e102449. doi: 10.2196/102449 (PMC13752591; doi:10.2196/102449)
Supplement: Supplementary file 2 [file formative_v10i1e102449_app2.pdf]

Multimedia Appendix 2. Example of an anonymized and translated student–avatar dialogue with the corresponding automated feedback

The dialogue below was conducted in Dutch in one of the pediatric cases. The case description shown to the student is given first. The student's utterances were captured by speech recognition. Obvious transcription errors were corrected and the text was edited for grammar, while staying as close as possible to the original wording. The dialogue and the feedback were translated from Dutch into English. The feedback is reproduced as generated by the system; it is written from the perspective of the simulated parent. No personal data of the student are included.

Case

Lotte (8 years old) has frequently recurring throat infections (7 times in the past year). The general practitioner refers her to the pediatrician to discuss a tonsillectomy. Her mother and father are divorced and in conflict. Today, you see her father, Bert, at the consultation.

Dialogue

Student: Good afternoon, can you hear me? Hello, can you hear me?

Avatar (father): Good afternoon. Yes, I'm here.

Student: What can I help you with today?

Avatar (father): I'm here for my daughter Lotte. She often suffers from throat infections, which has a big impact on her daily life. The general practitioner referred us to discuss a possible procedure.

Student: Okay. Can you tell me what the general practitioner has already explained, or what you already know yourself about the throat infections and the possible treatments?

Avatar (father): The general practitioner briefly explained that surgery is sometimes necessary if the throat infections keep coming back and affect her life. I have also looked up some of the pros and cons of removing the tonsils myself, but I would like to hear your advice on this.

Student: Okay, I certainly want to help you with this, but it is important for me to first ask a few questions to see what is important to you, because we will have to make this choice together: you, me, and also your daughter and her mother.

Avatar (father): I completely understand. Feel free to ask your questions; of course we want to make the best choice for Lotte.

Student: How often has Lotte had throat infections recently, in the past year?

Avatar (father): I think she has had a throat infection at least six or seven times this year. It varies a bit, but it really happens too often.

Student: Okay, and... one moment please, I'm just thinking.

Avatar (father): Of course, take your time.

Student: And do you already know which options there are for Lotte regarding the throat infections? Do you have an idea about that?

Avatar (father): Yes, I know there are usually two options: either we try medication for a while longer and hope it gets better on its own, or we have her tonsils removed if it really happens too often and bothers her too much. But I don't know exactly what would be most suitable in her situation.

Student: Okay, that is indeed a good question. It is as you say: there are two options. Either the tonsils are removed. That is done in children who, like Lotte, very often have recurrent throat infections, that is, infections of the tonsils. The operation is done under general anesthesia and the tonsils are removed, and afterwards the chance that she will have much trouble with them is much smaller. On the other hand, there is of course also the option to wait and simply treat the throat infections with medication when they come, as has been done so far, with painkillers and, if necessary, antibiotics. The advantage is that no anesthesia and no surgery are needed. Of course, it is then possible that she will get more throat infections, which can also bother her.

Avatar (father): Yes, that sounds logical. I understand there is something to be said for both options. What I'm wondering: how often do you have to have problems before such an operation is recommended? Is there some kind of guideline?

Student: I understand.

Avatar (father): Thank you. It's nice to hear that you explain this well. It really helps me get a better picture.

Student: Okay, you still had the question of when it is recommended. In principle, both options are equivalent: removing the tonsils, and simply waiting and treating the throat infections with painkillers. Both are equivalent; there is no better or worse option. Removal of the tonsils is usually done in children who indeed have it several times a year, say once every two months, so from about six or seven times a year, and when Lotte is also bothered by it, for example when she misses school because of it or there are other consequences. Then it can be recommended. But the other option, not having the operation, is medically speaking certainly also a valid option. It depends on what weighs most heavily for you.

Avatar (father): That sounds clear, thank you for the explanation. It is indeed the case that she has already missed school quite often because of those throat infections, and that also makes it difficult for us in terms of planning and childcare. But I do want to be sure that an operation is really necessary and that we think about it carefully.

Student: Yes. If you now weigh these two options, what would make you lean toward an operation? Which things play a role for you that would make you prefer an operation?

Avatar (father): What matters to us is mainly the impact on her life. She misses school, often feels unwell, and every time we have to use antibiotics and painkillers. That is not ideal. So if an operation can make her ill less often and allow her to pick up her normal life again, that would certainly be an important reason to choose it. But we do want to be sure that it is really necessary and that the advantages outweigh the disadvantages.

Student: Yes, I fully understand. So if I understand correctly, the important factors for you are the impact of missing school and often not feeling well, and also that it is difficult for you to plan in terms of childcare. Those are important factors for you?

Avatar (father): Yes, exactly. Those are certainly important factors for us. It simply has a lot of influence on her and on our family when she is ill so often.

Student: Yes, I can fully imagine that. Now, the operation has a possible risk of post-operative bleeding; that is the most important risk of the operation. It is an operation that is performed often, and Lotte would also have to undergo general anesthesia; she is put to sleep for the procedure. So those are more or less the most important risks. But of course, once the tonsils have been removed, we can say with reasonable certainty that she will have much less trouble, so the complaints will certainly decrease. So in the long run, that will certainly be an improvement in her daily life.

Avatar (father): That sounds positive, but I understand there are also risks. Post-operative bleeding does sound serious; that does worry me a bit. How often does that actually occur?

Student: It is certainly understandable that you are worried about that. I will quickly look up the figures on how often post-operative bleeding occurs. In the meantime, do you have any other questions about the option of waiting? As you already said, that means treatment with painkillers and antibiotics. Antibiotics also have side effects: the more antibiotics she takes, the greater the chance of resistance and of stomach and bowel problems. So that is also a factor that weighs in. Of course, you then do not have the risk of post-operative bleeding or of being put to sleep, which is still a drastic event for some children.

Avatar (father): Yes, I understand. That risk of resistance due to antibiotics is indeed also something we have to take into account. It's good that you explain it so clearly. I think we mainly need to look at what is best for Lotte in the long term.

Student: I have looked up the figures for you. After removal of the tonsils, 5 to 20 in 1000 children get post-operative bleeding. Post-operative bleeding is not necessarily serious, but in some cases the child has to be readmitted and have another operation to stop the bleeding. But that only happens in 5 to 20 in 1000 cases, so in the other 980 or so out of 1000 cases, there is actually no post-operative bleeding. So the risk is actually not that high.

Avatar (father): Ah, okay, that is good to know. So the risk is relatively small, but still something to take into account. Thank you for those figures. It helps us get a better picture of what the operation involves.

Student: Do you think that you already have a choice or a preference for one of the two options at this point: going ahead with an operation or not?

Avatar (father): I think we now have a better picture, but it remains a difficult choice. At the moment, I do lean more toward the operation, because it seems that it can give Lotte the most improvement in the long term. But I would still like to discuss it with my wife and also make sure that Lotte understands it herself.

Student: Yes, that indeed seems a good idea, to discuss it calmly a bit more. It is certainly not an urgent choice. It is also important that Lotte is well informed and that your whole family is on the same wavelength. I would suggest scheduling a follow-up appointment with Lotte and your wife, so that we can go over the options again. Is that something you would be open to?

Avatar (father): Yes, that seems like a very good idea. Then we can first discuss it properly at home and afterwards go over everything with you again. Thank you for the suggestion.

Student: Okay, then I will also give you the option grid. That is a decision aid on which both options are listed once more, with the pros and cons of each option, and you can go through it at home before you come to the follow-up appointment. Okay?

Avatar (father): That's very nice, thank you. Such an option grid will certainly help us put everything clearly in order. I appreciate that you guide us so well in this.

Student: Okay, no problem. Then we will see each other at the follow-up appointment.

Avatar (father): Great, thank you for your time and explanation today. We'll see each other again soon. Have a nice day!

Automated feedback

The conversation provides sufficient material to give feedback. Below is my assessment based on the six phases:

Phase 1: Creating clarity on the diagnosis

Positive points:

- You asked what I already knew about the diagnosis and treatment options, which allowed you to assess my prior knowledge.
- You explained the diagnosis and the options clearly, including the link between recurrent throat infections and the possible need for an operation.

Points for improvement:

- Although you clearly explained what is going on, you did not explicitly ask me to summarize in my own words what I understood about the diagnosis and the treatment options. This would help to check whether everything was clear.

Phase 2: Opening the conversation – creating a shared frame

Positive points:

- You made clear that a decision needs to be made and that I, as a parent, am involved in that decision.
- You created an open and inviting atmosphere by emphasizing that we will make a choice together.

Points for improvement:

- You mentioned the shared decision making, but you could have stated more explicitly that there are several options and that there is no “good” or “bad” choice. This would have given more room to think freely without pressure.

Phase 3: Information exchange – explaining the options

Positive points:

- You discussed both options (surgery and watchful waiting) clearly and extensively, including the advantages and disadvantages.
- You gave concrete information about risks, such as the chance of post-operative bleeding after surgery, and explained what this means in practice.
- You were patient and gave me room to ask questions.

Points for improvement:

- You did not explicitly check whether I understood the explanation of the options correctly, for example by asking me to summarize the pros and cons in my own words. You could improve this by applying the teach-back method.

Phase 4: Exploring values and preferences

Positive points:

- You actively asked what is important to us and showed understanding for the impact of the situation on our family.
- You checked whether you had understood my concerns correctly by summarizing the factors that are important (such as school and daily life).
- You took into account the role of my wife and daughter in the decision making.

Points for improvement:

- Although you listened well to what is important, you could have asked more about any specific concerns regarding the operation or watchful waiting. This would help to understand my preferences even better.

Phase 5: Decision making – arriving at a choice together

Positive points:

- You summarized the conversation and gave room to first discuss the choice at home with my family.

- You proposed a follow-up appointment to discuss the decision further together, which fits well with shared decision making.

Points for improvement:

- Although you summarized the options and preferences, you could have explicitly asked whether you understood my preference correctly and explained more about how we could make the final choice.

Phase 6: Closing

Positive points:

- You made clear that the choice is not final and that we can come back to it later.
- You provided practical information, such as an option grid, to discuss further at home.
- You closed the conversation in a friendly and professional manner.

Points for improvement:

- You could have explicitly asked whether I had any other questions before closing the conversation. This would have given me an extra opportunity to discuss any remaining doubts or uncertainties.

General communication skills

Positive points:

- You used understandable language and gave detailed explanations without complicated terms.
- You showed empathy and listened well to my concerns, which made me feel heard and understood.
- You created a safe atmosphere by being inviting and patient.

Points for improvement:

- Although you were clear and patient, you could have made more use of reflective listening, for example by naming my concerns and emotions more explicitly ("I hear that you are worried about the risks; that is very understandable.").

Summary and suggestions

Overall, this was a good conversation. You gave clear information, involved me in the decision making and left room for questions and consultation. To make it even better, you can:

- Apply the teach-back method more often to check whether I understood everything.
- Ask further about specific concerns and preferences regarding the options.
- Use more reflective listening to name my emotions and concerns more explicitly.
- Close the conversation more systematically by explicitly asking about any remaining questions.
