# Supplementary material for: Large Language Model–Powered 3D Avatars to Train Shared Decision-Making in Undergraduate Medical Students: Design and Mixed Methods Acceptance Study
Source: JMIR Form Res. 2026 Oct 27;10:e102449. doi: 10.2196/102449 (PMC13752591; doi:10.2196/102449)
Supplement: Supplementary file 3 [file formative_v10i1e102449_app3.pdf]

Multimedia Appendix 3. Comparison of construct scores between students who practiced with pediatric cases (n=11) and those who practiced with orthopedic cases (n=13).

**Construct**	**Pediatric M (SD)**	**Orthopedic M (SD)**	**p**
Perceived usefulness	4.545 (0.402)	4.333 (0.561)	.33
Perceived ease of use	3.879 (0.522)	3.744 (0.807)	.81
Intention to use	4.394 (0.512)	4.436 (0.672)	.66
